# Use of Vedolizumab in Inflammatory Bowel Disease: A Single-Center Experience

**DOI:** 10.5152/tjg.2022.21684

**Published:** 2022-10-01

**Authors:** Çağdaş Erdoğan, Bayram Yeşil, Ferhat Bacaksız, Derya Arı, Volkan Gökbulut, Mahmut Yüksel, Yasemin Özderin Özin, Ertuğrul Kayaçetin

**Affiliations:** 1 Department of Gastroenterology, Ankara City Hospital, Ankara, Turkey

**Keywords:** Crohn’s disease, inflammatory bowel disease, ulcerative colitis, vedolizumab

## Abstract

**Background:**

Vedolizumab, which is a monoclonal antibody that selectively binds to α4β7 integrin in the gastrointestinal system, may be an effective and safe treatment alternative in those with anti-tumor necrosis factor-resistant inflammatory bowel disease.

**Methods:**

Patients administered vedolizumab due to anti-tumor necrosis factor resistant or anti-tumor necrosis factor side effects between August 2017 and November 2020 were included in the study. Crohn’s patients were evaluated using the Harvey–Bradshaw index and Simple Endoscopic Score for Crohn’s Disease, whereas ulcerative colitis patients were evaluated with the Partial Mayo Score Index and Rachmilewitz score. All patients were followed up for 3 months and their blood samples were taken every 3 months. Hemoglobin, white blood cell, leukocyte, lymphocyte, and platelet counts of the patients were performed. Albumin, C-reactive protein, and erythrocye sedimentation rate values were recorded. The side effect profile for vedolizumab was evaluated for all patients. Among the side effects, arthralgia and flu-like symptoms were observed.

**Results:**

A total of 48 patients (18 ulcerative colitis and 30 Crohn’s disease) were included in the study. Vedolizumab therapy was initiated in the patients due to anti-tumor necrosis factor resistance (17 ulcerative colitis and 26 Crohn’s disease) or anti-tumor necrosis factor side effects (1 ulcerative colitis and 4 Crohn’s disease). A total of 30 (63%) patients, including 15 (83%) ulcerative colitis and 15 (50%) Crohn’s disease, responded to treatment (both response and remission). The mean duration of response to treatment was 4.5 ± 1.5 months. A total of 20 (42%) patients in the vedolizumab therapy subgroup (10/10, ulcerative colitis/Crohn’s disease) went into remission. The mean Harvey–Bradshaw Index value was 9.8 ± 2.8 in the Crohn’s disease patients at the time of initial treatment. The mean Simple Endoscopic Score for Crohn’s disease value was 11.2 ± 3.1 at the time of initial treatment. The mean Harvey–Bradshaw Index value was 6.5 ± 3.0 and the mean Simple Endoscopic Score for Crohn’s disease value was 4.9 ± 3.6 at 6 months post-treatment. The mean Ulcerative Colitis Endoscopic Index (Rachmilewitz) value was 9.3 ± 1.2 at the time of initial treatment. In addition, the mean Partial Mayo Scoring Index was 6.4 ± 1.5 at the time of initial treatment. The mean Ulcerative Colitis Endoscopic Index (Rachmilewitz) value was 0 (0-6.0), and the mean Partial Mayo Scoring Index was 1.5 (0.3-4.0) at 6 months post-treatment.

**Conclusion:**

Vedolizumab therapy is effective in both induction and maintenance of remission in inflammatory bowel disease patients who are resistant to anti-tumor necrosis factor or who can not receive anti-tumor necrosis factor therapy due to side effects. No significant side effect was observed in the patients during follow-up.

**Main Points**

- Vedolizumab can be used in remission induction in tumor necrosis factor-resistant inflammatory bowel disease patients.
- The most severe patients will be the most responsive ones.
- The side effect profile of vedolizumab is quite safe.
- The patients who did not respond to the treatment could develop side effects.

## Introduction

Ulcerative colitis (UC) and Crohn’s disease are 2 major groups of inflammatory bowel diseases (IBDs). Ulcerative colitis mostly progresses with colon involvement, whereas Crohn’s disease may be seen in the entire gastrointestinal system. Inflammatory bowel diseases are immune-mediated inflammatory diseases with relapses and are caused by the combination of immune, microbial, and genetic factors.^1,2^ Anti-inflammatory drugs such as aminosalicylates and corticosteroids, immunosuppressant drugs such as thiopurines and methotrexate, and anti-tumor necrosis factor (anti-TNF) drugs are used in the treatment of IBDs. Anti-TNF treatments can be used in remission induction and maintenance therapy. These treatments have been found to have an effect on mucosal improvement and to decrease hospitalization, and the need for surgery and steroids.^3^ However, primary unresponsiveness is seen in 30% and secondary response loss in 50% of all patients.^4^ In addition, patients who previously received another anti-TNF drug had a lower response to the second anti-TNF drug.^5^ Therefore, vedolizumab may stand out in anti-TNF-resistant, steroid-unresponsive, or dependent patients as a treatment alternative with different mechanisms of action.

Vedolizumab is a human-origin monoclonal immunoglobulin G-1 antibody, which selectively binds to α4β7 integrin that is a cell-surface glycoprotein in the gastrointestinal tract and inhibits binding of this with mucosal adhesion molecule 1. In this way, lymphocyte translocation from the bloodstream to the inflamed intestine is prevented.^6,7^

The efficacy of vedolizumab was evaluated in the randomized controlled, double-blind, placebo-controlled GEMINI studies, and it was licensed for the treatment of patients with moderate-to-severe UC and Crohn’s disease.^8,9^

## Materials and Methods

The patients who were administered vedolizumab (Entyvio; Takeda) treatment between August 2017 and November 2020 were included in our study. Patients’ demographic data such as age, gender, and smoking history were questioned. In addition, patients’ medical histories were received and whether they had hypertension and diabetes was recorded. The dates of starting vedolizumab treatment and follow-up periods of all patients were evaluated. The dates of the diagnosis of IBD and follow-up periods were also evaluated.

The involvement patterns of the patients for Crohn’s disease were evaluated as ileal disease, ileocolonic disease, and colonic disease. The presence of perianal disease was evaluated and recorded. Disease behaviors for Crohn’s disease were recorded as inflammatory, stricturing, penetrating, and their binary combinations.

Disease involvement for UC was evaluated as left-type colitis, extensive colitis, or pancolitis.

Initiation indications were evaluated in the patients who started to receive vedolizumab therapy. Accordingly, the patients were assessed as those who can not use anti-TNF due to resistance or side effects due to anti-TNF use. It was evaluated whether the patients had single or dual anti-TNF resistance.

Surgical histories of the patients and if surgery was performed, the surgical procedures performed were recorded.

The Crohn’s patients were evaluated using Harvey–Bradshaw Index (HBI) and Simple Endoscopic Score for Crohn’s disease (SES-CD), and the UC patients with Partial Mayo Scoring (PMSI) and Rachmilewitz at 0, 3, 6, 12, 24, and 36 months of follow-up, according to the follow-up duration.

In the response evaluation, remission for Crohn’s disease was determined as an HBI < 5 and SES-CD ≤ 2. The response criterion for UC was determined as a PMSI < 2 and Rachmilewitz < 4.

For Crohn’s disease, HBI > 4 or HBI ≤ 4 and endoscopic stenosis and/or mucosal damage were accepted as the active disease. Again for Crohn’s disease, SES-CD >2 was accepted as the active disease. For UC, PMSI ≥ 2 or Rachmilewitz ≥ 4 was evaluated as an active disease.

For Crohn’s disease patients, patients with a decrease in HBI and SES-CD according to baseline scores and who did not meet the remission criteria were accepted to have a response. Similarly, for UC disease, patients with a decrease in PMSI and Rachmilewitz scores and who did not meet the remission criteria were accepted to have a response.

All patients were followed up for 3-month periods and their blood samples were taken every 3 months. Patients’ hemoglobin, white cells, leukocytes, lymphocytes, and platelets were counted; albumin, C-reactive protein, and erythrocyte sedimentation rate values were recorded.

All patients’ side effect profiles for vedolizumab were evaluated. Among the side effects, arthralgia and flu-like symptoms were detected.

The treatments that the patients received before vedolizumab were recorded. Accordingly, patients who received 5-acetylsalicylic acid, azathioprine, methotrexate, steroids, and those who received anti-TNF were evaluated. Patients who received anti-TNF prior to vedolizumab were individually evaluated as those who received adalimumab only, those who received infliximab only, or both during their treatment.

The study was conducted with the approval of the Clinical Research Ethics Committee (approval no: E1-20-1214). We took the consent of all patients by asking them to fill in informed consent forms before the study.

### Statistical Analysis

Statistical analysis was performed with Statistical Package for the Social Sciences (SPSS) 23.0 software (IBM Corp.; Armonk, NY, USA). The normality of the variables and groups was evaluated through Kolmogorov–Smirnov test. Independent sample *t*-test and Mann–Whitney *U* tests were used in the comparison of the variables expressed as mean ± standard deviation and non-parametric quantitative variables, respectively. Categorical variables were compared with Pearson’s chi-square test, but Fisher’s exact test was performed when 2 of the expected values were below 5 or 1 of the expected values was below 2. Log-rank test was used to evaluate response to treatment during follow-up. Two-tailed *P* < .05 values were considered statistically significant.

## Results

A total of 52 patients were given vedolizumab during the study period. Two patients were excluded from the study because their starting time of treatment was less than 3 months and 2 patients because they were already in remission at the beginning of the treatment. All patients included in the study were active patients clinically and endoscopically.

A total of 48 patients (18 UC and 30 Crohn’s disease) were included in the study. The median duration of follow-up was 10.0 (6.0-17.8) months for all patients. Baseline characteristics and laboratory parameters of the study population are summarized in Table 1. Vedolizumab therapy was initiated in the patients due to anti-TNF resistance (17 UC and 26 Crohn’s disease) or anti-TNF side effects (1 UC and 4 Crohn’s disease). A total of 30 patients (63%), including 15 (83%) UC and 15 (50%) Crohn’s disease patients responded to treatment (both response and remission). The mean duration of response to treatment was 4.5 ± 1.5 months. Twenty patients in the vedolizumab therapy subgroup (42%) (10/10, UC/Crohn’s disease) went into remission. Five patients had side effects (2 arthralgia and 3 flu-like symptoms). While 32 (67%) of the patients (UC/Crohn’s disease, 16/16) had previously received steroid treatment, 23 (48%) (UC/Crohn’s disease, 6/17) patients had undergone surgery.

**Figure 1.**
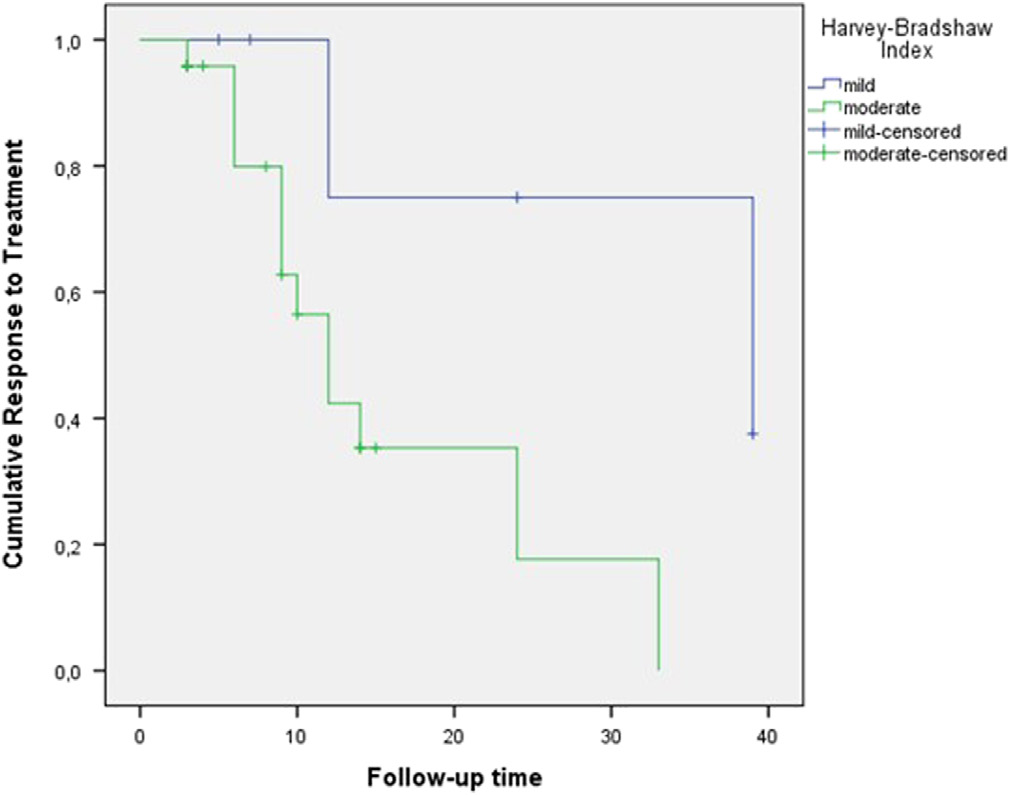
Treatment response according to Harvey–Bradshaw Index in patients with Crohn’s disease.

The mean HBI value in Crohn’s patients was 9.8 ± 2.8 at the time of initial treatment (Table 2). Based on HBI, 6 patients had mild and 24 had moderate disease, while none of the patients had severe disease. During the follow-up period, response to treatment was better in the patients with moderate disease according to HBI (*P* = .032) (Figure 1). The mean SES-CD value was 11.2 ± 3.1 at the time of initial treatment. Based on SES-CD, 12 patients had mild and 18 had moderate disease, while none of the patients had severe disease.

**Figure 2.**
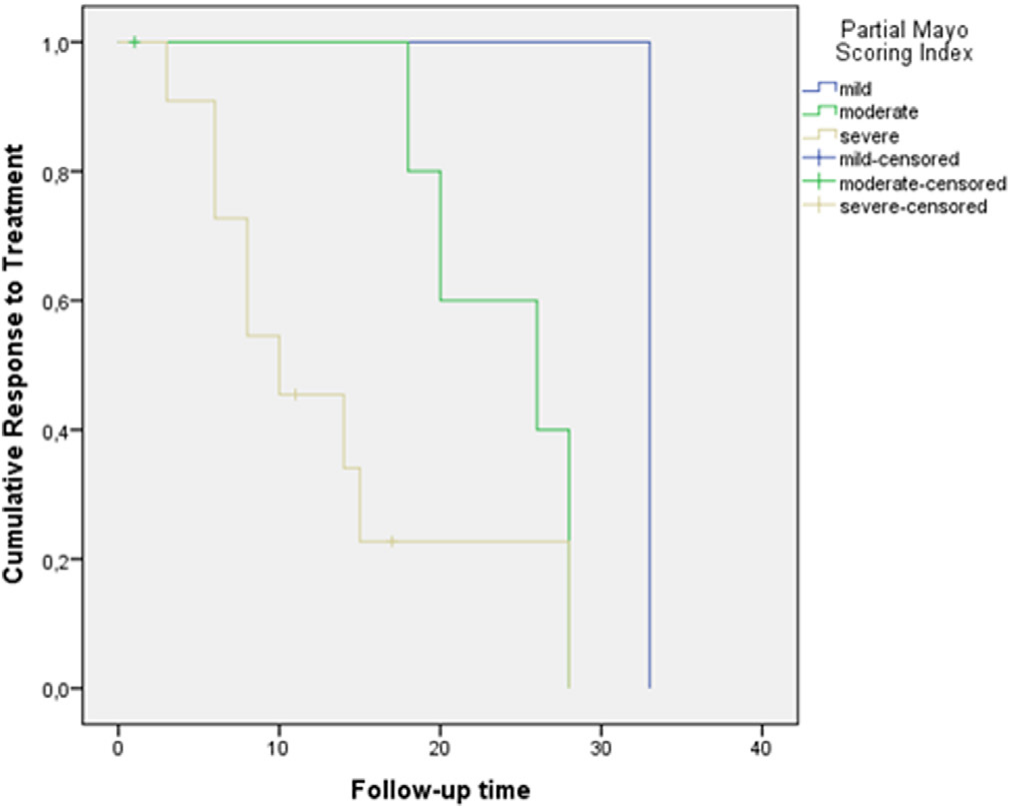
Treatment response according to Partial Mayo Scoring Index in patients with ulcerative colitis.

The mean Ulcerative Colitis Endoscopic Index (Rachmilewitz) value was 9.3 ± 1.2 at the time of initial treatment (Table 3). In addition, the mean PMSI value was 6.4 ± 1.5 at the time of initial treatment. Based on PMSI, 1 patient had mild, 6 patients had moderate, and 11 patients had severe disease. During the follow-up period, response to treatment was better in the patients with the severe disease according to PMSI (*P* = .012) (Figure 2).

Response to treatment was better in UC patients compared to Crohn’s disease patients (*P* = .02). Side effects were less in patients who responded to treatment (*P* = .02), while all other characteristics were similar in both groups (UC and Crohn’s disease) (Table 4). In addition, baseline clinical and laboratory parameters according to remission are presented in Table 5. No significant difference was found between the patients with and without remission in terms of clinical and laboratory features (for all *P *> .05). Treatment and response to treatment of the patients with Crohn’s disease and UC subtypes are presented in Table 6 in detail.

## Discussion

Vedolizumab therapy is indicated in the treatment of moderate or severe UC and Crohn’s disease worldwide, whereas in our country, vedolizumab is a treatment alternative in IBD patients refractory to anti-TNF treatments or who can not receive these treatments due to side effects. In our study, vedolizumab therapy was administered in patients resistant to anti-TNF therapy or those who cannot use anti-TNF because of the mentioned reasons, and responses to treatment were evaluated. In a study by Sands et al^10^ on Crohn’s patients, anti-TNF refractory patients were shown to benefit from vedolizumab therapy after 10 weeks. In our study, the first response evaluation was made at 3 months and response and remission were obtained in anti-TNF resistant patients.

Vedolizumab is indicated in moderate and severe UC and Crohn’s disease. In the GEMINI 1 study conducted by Feagan et al^8^ the remission rate was found to be 47.1% in the induction treatment reached in 6 weeks in UC patients. In the same study, the remission maintenance rate was 44.1%. In our study, UC patients were evaluated with PMSI and Endoscopic Activity Index (EAI) at 3 months, and the response rate was found as 83% and remission rate as 56%. During follow-up, remission was maintained in 50% of the patients. Remission disappeared in 1 patient who developed secondary loss of response later.

In the GEMINI 2 study performed by Sandborn et al^9^ remission rate reached at 6 weeks was reported as 14.5% and the remission continued in 39% of the patients. In our study, Crohn’s disease patients were evaluated with HBI and SES-CD scores at 3 months and the response rate was found as 50% and remission rate as 33%.

Consistent with both studies, vedolizumab was found to be effective in both remission induction and maintenance therapy in patients with Crohn’s disease and UC. Considering the subgroup analyses, remission induction rates were found to be higher in the treatment of UC than Crohn’s disease, and these rates are consistent with the literature.

In a study by Amiot et al.^11^ on 294 patients with anti-TNF refractory IBD, steroid-free remission was found in 31% and response to treatment in 50% of Crohn’s disease patients at 14 weeks, whereas, steroid-free remission was found in 36% and response to treatment in 50% of patients with UC. In our study, although similar remission and response rates were found for the Crohn’s disease patients, higher rates of remission and response were achieved in the UC patients.

In another study, Engel et al.^12^ reviewed 9 studies including real-life data and conducted efficacy and safety analysis of vedolizumab on 1565 IBD patients. In that study, response and remission rates were found as 49% and 32% for Crohn’s disease and 51% and 30% for UC at 14 weeks. In our study, while the rates of remission and response were similar for Crohn’s disease, these rates were higher for UC.

In a study by Colombel et al.^13^ with 2830 patients about the safety of vedolizumab in IBD patients, vedolizumab was found to be very safe, and the rate of serious side effects such as severe infection, infusion-related side effects, or malignancy due to long-term treatment was found to be quite low. In another study by Reinglas et al.^14^ with 130 IBD patients treated with vedolizumab, 3 patients developed arthralgia during follow-up. In our study, arthralgia was found in 2 (4.2%) and flu-like symptoms in 3 (6.3%) patients. Apart from that, none of our patients had a severe infection or infusion-related event that could be considered as serious side effects.

Another noteworthy finding of our study was that rates of response to vedolizumab therapy increased proportionally with the disease severity. Better responses were achieved with vedolizumab in IBD patients with moderate-to-severe disease scores. Similarly, involvement rates affected response to treatment, and higher rates were found in ulcerative patients with pancolitis and Crohn’s disease patients with ileocolitis.

The most important limitation of our study is the limited follow-up duration of the patients. In addition, the effectiveness of vedolizumab could not be evaluated in anti-TNF naïve patients due to the reimbursement conditions in our country.

## Conclusion

Vedolizumab therapy was effective in both induction and maintenance of remission in IBD patients who are anti-TNF resistant or who can not receive anti-TNF treatment due to side effects. The rates of response were higher in patients with UC compared to those with Crohn’s disease. The response rates to vedolizumab therapy increased as the disease severity increased. In addition, the pattern of involvement is another finding affecting the rates of response to vedolizumab. No serious side effect was observed in patients. Patients with side effects were mostly those who did not respond to treatment.

## Figures and Tables

**Table 1.** Baseline Clinical and Laboratory Parameters

	Total (n = 48)	Ulcerative Colitis (n = 18)	Crohn’s Disease (n = 30)
Age (years)	44.1 ± 12.9	47.3 ± 15.6	42.1 ± 10.8
Gender male, n (%)	28 (58)	12 (67)	16 (53)
Hypertension, n (%)	6 (13)	2 (11)	4 (13)
Diabetes, n (%)	1 (2)	1 (6)	0 (0)
Smoking, n (%)	23 (48)	8 (44)	15 (50)
Treatment response, n (%)	30 (63)	15 (83)	15 (50)
Treatment response time, months	4.5 ± 1.5	3.8 ± 1.4	5.2 ± 1.4
Remission, n (%)	20 (42)	10 (56)	10 (33)
Side effect, n (%)	5 (10)	0 (0)	5 (17)
Indication of vedolizumab, n (%) Anti-TNF resistance Anti-TNF side effect	43 (90) 5 (10)	17 (94) 1 (6)	26 (88) 4 (12)
Anti-TNF therapy, n (%) N/A Infliximab Adalimumab Infliximab + adalimumab	5 (10) 10 (21) 11 (23) 22 (46)	3 (17) 2 (11) 8 (44) 5 (28)	2 (7) 8 (27) 3 (10) 17 (57)
Perianal disease, n (%)	9 (20)	0 (0)	9 (30)
Follow-up time, months	10.0 (6.0-17.8)	14.5 (7.5-26.5)	9.0 (5.7-14.0)
Time since diagnosis, months	132.0 (85.7-177.8)	114.0 (89.2-173.2)	144.0 (84.0-180.0)
Steroid therapy, n (%)	32 (67)	16 (89)	16 (53)
Surgery, n (%)	23 (48)	6 (33)	17 (57)
Hemoglobin, g/dL	12.7 ± 0.9	12.3 ± 1.0	13.3 ± 0.5
Platelet, ×10^3^/mm^3^	319.9 ± 135.2	341.9 ± 183.1	304.6 ± 89.9
WBC, ×10^3^/mm^3^	7.8 ± 2.3	7.7 ± 2.9	7.8 ± 1.9
Neutrophil, ×10^3^/mm^3^	5.1 ± 1.7	4.4 ± 1.8	5.4 ± 1.6
Lymphocyte, ×10^3^/mm^3^	2.7 ± 0.7	2.0 ± 0.8	2.1 ± 0.6
Albumin, gr/dL	4.2 ± 0.5	4.2 ± 0.6	4.2 ± 0.4
CRP, mg/dL	8.7 (2.9-17.4)	13.3 (2.8-22.3)	6.7 (2.7-17.1)

Results are expressed as: mean ± SD or median (IQR) or frequency (%). TNF, tumor necrosis factor; WBC, white blood cell; CRP, C-reactive protein.

**Table 2.** International Scores for Crohn’s Disease

	At the Time of Initial Treatment	3 Months	6 Months	12 Months	24 Months	36 Months
HBI	9.8 ± 2.8	8.5 ± 3.7	6.5 ± 3.0	6.6 ± 4.1	-	-
SES	11.2 ± 3.1	-	4.9 ± 3.6	7.2 ± 6.2	-	-

Results are expressed as mean ± SD or median (IQR) or frequency (%). HBI, Harvey–Bradshaw Index; SES, The Simple Endoscopic Score.

**Table 3.** International Scores for Ulcerative Colitis

	At the Time of Initial Treatment	3 Months	6 Months	12 Months	24 Months	36 Months
PMSI	6.4 ± 1.5	3.8 ± 2.5	1.5 (0.3-4.0)	1.5 (0-3.8)	-	-
Ulcerative Colitis Endoscopic Index	9.3 ± 1.2	4.8 ± 3.8	0 (0-6.0)	-	-	-

Results are expressed as: mean ± SD or median (IQR) or frequency (%). PMSI, Partial Mayo Scoring Index.

**Table 4.** Baseline Clinical and Laboratory Parameters According to Treatment Response

	Treatment Response (+) (n = 30)	Treatment Response (−) (n = 18)	*P*
Age (years)	42.2 (12.7)	47.2 ± 12.9	.19
Gender male, n (%)	17 (57)	11 (61)	.76
Hypertension, n (%)	2 (7)	1 (6)	.18
Diabetes, n (%)	0 (0)	4 (22)	.19
Smoking, n (%)	16 (53)	7 (39)	.58
Crohn’s disease, n (%)	15 (50)	15 (83)	**.02**
Ulcerative colitis, n (%)	15 (50)	3 (17)	**.02**
Treatment response time, months	4.5 ± 1.5	N/A	N/A
Remission, n (%)	20 (67)	N/A	N/A
Side effect, n (%)	1 (3)	4 (22)	**.02**
Indication of vedolizumab, n (%) Anti-TNF resistance Anti-TNF side effect	26 (87) 4 (13)	16 (89) 2 (11)	1.00 1.00
Anti-TNF therapy, n (%) N/A Infliximab Adalimumab Infliximab + adalimumab	26 (87) 4 (13) 7 (23) 7 (23) 12 (40)	17 (94) 1 (6) 3 (17) 4 (22) 10 (56)	.64
Perianal disease, n (%)	3 (10)	6 (33)	.045
Follow-up time, months	12.0 (7.5-24.5)	8.5 (3.0-14.2)	.08
Time since diagnosis, months	114.0 (84.0-173.2)	150.0 (117.0-180.0)	.17
Steroid therapy, n (%)	20 (67)	12 (67)	1.00
Surgery, n (%)	15 (50)	8 (44)	.71
Hemoglobin, g/dL	12.7 ± 0.5	12.7 ± 1.2	1.00
Platelet, ×10^3^/mm^3^	302.4 ± 138.5	357.3 ± 124.5	.21
WBC, ×10^3^/mm^3^	7.8 ± 2.3	7.8 ± 2.5	.90
Neutrophil, ×10^3^/mm^3^	4.7 ± 1.5	6.3 ± 2.1	**.03**
Lymphocyte, ×10^3^/mm^3^	2.1 ± 0.6	1.9 ± 0.7	.48
Albumin, gr/dL	4.3 ± 0.4	4.1 ± 0.5	.15
CRP, mg/dL	9.0 (2.7-15.5)	7.5 (2.8-25.7)	.72

Results are expressed as: mean ± SD or median (IQR) or frequency (%). TNF, tumor necrosis factor; WBC, white blood cell; CRP, C-reactive protein.

**Table 5.** Baseline Clinical and Laboratory Parameters According to Remission

	Remission (+) (n = 20)	Remission (−) (n = 28)	*P*
Age (years)	40.7 ± 14.0	46.5 ± 11.6	.13
Gender male, n (%)	9 (45)	11 (39)	.69
Hypertension, n (%)	2 (10)	4 (14)	1.00
Diabetes, n (%)	0 (0)	1 (4)	1.00
Smoking, n (%)	12 (60)	11 (39)	.36
Crohn’s disease, n (%)	10 (50)	20 (71)	.13
Ulcerative colitis, n (%)	10 (50)	8 (29)	.13
Treatment response time, months	4.2 ± 1.5	5.1 ± 1.4	.13
Side effect, n (%)	1 (5)	4 (14)	.38
Indication of vedolizumab, n (%) Anti-TNF resistance Anti-TNF side effect	16 (80) 4 (20)	26 (93) 2 (7)	.70 .22
Anti-TNF therapy, n (%) N/A Infliximab Adalimumab Infliximab + adalimumab	18 (90) 2 (10) 6 (30) 2 (10) 10 (50)	25 (89) 3 (11) 4 (14) 9 (32) 12 (43)	1.00
Perianal disease, n (%)	2 (10)	7 (25)	.19
Follow-up time, months	12.0 (6.7-27.5)	9.0 (3.2-14.7)	.08
Time since diagnosis, months	108.0 (84.0-132.0)	156.0 (111.0-180.0)	**0.02**
Steroid therapy, n (%)	8 (40)	8 (29)	.41
Surgery, n (%)	12 (60)	11 (39)	.16
Hemoglobin, g/dL	12.5 ± 0.7	12.8 ± 0.9	.68
Platelet, ×10^3^/mm^3^	306.2 ± 99.8	331.3 ± 160.2	.55
WBC, ×10^3^/mm^3^	8.1 ± 2.2	7.6 ± 2.5	.67
Neutrophil, ×10^3^/mm^3^	4.9 ± 1.9	5.2 ± 2.1	.71
Lymphocyte, ×10^3^/mm^3^	2.1 ± 0.5	2.0 ± 0.7	.58
Albumin, gr/dL	4.3 ± 0.3	4.1 ± 0.5	.40
CRP, mg/dL	7.6 (2.3-16.5)	8.7 (3.0-18.6)	.68

Results are expressed as: mean ± SD or median (IQR) or frequency (%). TNF, tumor necrosis factor; WBC, white blood cell; CRP, C-reactive protein.

**Table 6.** Subtypes of Inflammatory Bowel Diseases

	Total	Treatment Response (+)	Treatment Response (−)	Remission (+)	Remission (−)
Crohn’s disease, total n (%) Ileitis Ileocolitis Colitis	30 5 (17) 20 (67) 5 (17)	15 2 (13) 13 (87) 0 (0)	15 3 (20) 7 (47) 5 (33)	10 1 (10) 9 (90) 0 (0)	20 4 (20) 11 (55) 5 (25)
Crohn’s disease, total, n (%) Stricture Penetrating Inflammatory Penetrating + stricture Penetrating + inflammatory İnflammatory + stricter	30 3 (10) 2 (7) 13 (4) 2 (7) 5 (17) 3 (10)	19 2 (11) 1 (5) 6 (32) 1 (5) 2 (11) 3 (16)	13 0 (0) 1 (8) 7 (54) 1 (8) 3 (23) 1 (8)	10 2 (20) 1 (10) 2 (20) 1 (10) 1 (10) 3 (30)	19 1 (5) 1 (5) 11 (58) 1 (5) 4 (21) 1 (5)
Ulcerative colitis, total n (%) Left-sided colitis Pancolitis Extensive colitis	18 5 (28) 9 (50) 4 (22)	15 5 (33) 8 (53) 2 (13)	3 0 (0) 1 (33) 2 (66)	10 2 (20) 6 (60) 2 (20)	8 3 (38) 3 (38) 2 (25)

Results are expressed as frequency (%).
